# Composición corporal, aptitud física y entrenamiento físico en escolares de un colegio público

**DOI:** 10.15446/rsap.V25n4.99605

**Published:** 2023-07-01

**Authors:** Nohora E. Alvarez-Rey, Mónica A. Amador-Ariza, Jhoalmis Sierra-Castrillo, Paul A. Camacho-López

**Affiliations:** 1 NA: FT. Esp. Gerencia y Auditoría de la Calidad en Salud, Facultad de Ciencias Médicas y de la Salud, Universidad de Santander. Cúcuta, Colombia, no.alvarez@mail.udes.edu.co Universidad de Santander Facultad de Ciencias Médicas y de la Salud Universidad de Santander Cúcuta; 2 MA: FT. Esp. Gestión de Proyectos, Facultad de Ciencias Médicas y de la Salud, Universidad de Santander. Cúcuta, Colombia. moni.amador@mail.udes.edu.co Universidad de Santander Facultad de Ciencias Médicas y de la Salud Universidad de Santander Cúcuta Colombia; 3 JS: BACT. M. Sc. Bioquímica Clínica. Facultad de Ciencias Médicas y de la Salud, Universidad de Santander. Cúcuta, Colombia. jho.sierra@mail.udes.edu.co Universidad de Santander Facultad de Ciencias Médicas y de la Salud Universidad de Santander Cúcuta Colombia; 4 PC: MD. M. Sc. Epidemiología. Fundación Oftalmológica de Santander. Bucaramanga, Colombia. paul.camacho@foscal.com.co Fundación Oftalmológica de Santander Fundación Oftalmológica de Santander Bucaramanga Colombia

**Keywords:** Ejercicio físico, adolescente, antropometría, aptitud física, índice de masa corporal *(fuente: DeCS, BIREME)*, Physical exercise, adolescent, anthropometry, physical fitness, body mass index *(source: MeSH, NLM)*

## Abstract

**Objetivo:**

Describir la composición corporal, la aptitud física y los marcadores de riesgo bioquímico pre y post implementación de un programa de ejercicio físico en adolescen tes de 10 a 17 años de un colegio público.

**Metodología:**

Estudio con enfoque experimental, la muestra estuvo conformada por 276 escolares, [141] grupo intervención y [135] grupo control. Se realizó el diligenciamiento del consentimiento y asentimiento informado, se tomaron medidas antropométricas, las pruebas de la batería Fitnessgram y se implementó un programa de ejercicio físico.

**Resultados:**

Después de la implementación del programa de ejercicio físico se reportó aumento de peso y perímetro de cintura en niñas. Los escolares de 10 a 13 años pre sentaron una disminución del colesterol y triglicéridos. Los hombres presentaron mejor rendimiento en las pruebas de la batería Fitnessgram, la capacidad aeróbica y la fuerza. En la prueba Trunk Lift y Sit and reach, las mujeres exhibieron mayor flexibilidad.

**Conclusiones:**

Es importante evaluar la condición física de los escolares como estra tegia para la prevención de enfermedades crónicas no trasmisibles. A su vez, se reco mienda que se promueva la práctica regular de actividad física en el entorno escolar y en el hogar como factor protector de la salud.

## Abstract (en)

**Objective:**

To describe the body composition, physical fitness and biochemical risk markers before and after implementation of a physical exercise program in adolescents aged 10 to 17 years of a public school.

**Methodology:**

Study with an experimental approach, the sample consisted of 276 schoolchildren, (141) intervention group and (135) control group. Informed consent and assent were completed, anthropometric measurements were taken, the Fitnessgram battery tests were carried out, and a physical exercise program was implemented.

**Results:**

Post implementation of the physical exercise program, weight gain and waist circumference were reported in girls. Schoolchildren from 10 to 13 showed a decrease in cholesterol and triglycerides. Men performed better in the Fitnessgram battery, aerobic capacity and strength tests. In the Trunk Lift, and Sit and Reach tests, women exhibited greater flexibility.

**Conclusions:**

It is important to evaluate the physical condition of schoolchildren, as a strategy for the prevention of chronic non-communicable diseases. In turn, it is recommended that the regular practice of physical activity be promoted in the school and home environment, as a protective factor of health.

La aptitud física es el conjunto de atributos que la persona tiene y que se relacionan con la habilidad para llevar a cabo actividades de la vida diaria con vigor, alerta y sin fatiga. Incluye cuatro componentes: composición corporal, resistencia cardiorrespiratoria, flexibilidad, fuerza y resistencia muscular 1.

La condición física (CF) es un importante indicador fisiológico del estado de salud de la población y un predictor de morbimortalidad por enfermedad cardiovas cular, sobrepeso y obesidad; en los últimos años, se ha reportado un alarmante descenso en los niveles de la CF de los niños y los adolescentes 2.

La inactividad física es uno de los problemas de salud más urgentes que requieren solución en siglo XXI, ya que contribuye a la aparición de enfermedades crónicas no transmisibles (ECNT), enfermedades mentales y dismi nución del rendimiento escolar. Colombia es el sexto país en Latinoamérica con mayores índices de mortalidad por causa de la inactividad física, como consecuencia del desarrollo tecnológico experimentado en los últimos años, el cual ha generado un cambio de estilo de vida de la población mundial, con una reducción importante de la actividad física. Esto tiene un impacto negativo en la salud y la calidad de vida 3.

En 2016 más de 340 millones de niños y adolescentes (de 5 a 17 años) presentaron sobrepeso u obesidad. La prevalencia del sobrepeso y la obesidad en niños y adolescentes ha aumentado de forma exponencial, del 4% en 1975 a más del 18% en 2016. Este aumento ha sido similar en ambos sexos: 18% de niñas y 19% de niños con sobrepeso en 2016. Según el Fondo de las Naciones Unidas para la Infancia (Unicef, por su sigla en inglés) y la Organización Mundial de la Salud (OMS), en la actualidad, el 33,6% de los niños, niñas y adoles centes está afectado por sobrepeso u obesidad. El 81% de los adolescentes del mundo es sedentario; la inactividad física impacta negativamente en la aptitud física y por ende en la composición corporal 4.

Según la Encuesta Nacional de Situación Nutricional 2015, siete de cada diez escolares y ocho de cada diez adolescentes (76,6%) pasan tiempo excesivo frente a pantallas (dos horas o más al día); se reportaron mayores proporciones en las zonas urbanas, Bogotá tiene la mayor prevalencia de conducta sedentaria en todos los grupos de edad. El tiempo excesivo frente a pantallas se asocia con una composición corporal menos favorable, mayores puntajes en indicadores de riesgo metabólico, una condición física inferior y puntajes bajos en indicadores de salud emocional y social 5.

La alta carga de sobrepeso y obesidad infantil presenta graves consecuencias para la salud, la sociedad y la economía, afecta a las familias, las comunidades y el sistema de salud a corto y largo plazo. Por tal motivo, la prevención de la obesidad y el sobrepeso en etapas tempranas son responsabilidad del Gobierno, la sociedad civil, el sector privado, las comunidades y las familias. Para cumplir este objetivo es necesario transformar los entornos, ya que en Colombia la intensidad horaria del área de educación física para los jóvenes es de dos horas semanales; además, es fundamental hacer un análisis de la composición corporal en los escolares, promover la actividad física y una adecuada nutrición en edades tempranas como factor protector 6.

La actividad física es cualquier movimiento corporal voluntario, repetitivo que involucra grandes grupos musculares y aumenta el gasto energético por encima del nivel de reposo; genera múltiples beneficios en los diferentes sistemas tales como el control de peso, el aumento de la densidad ósea y la fuerza muscular; la mejora la coordinación, aumenta la autoestima, mejora el autoconcepto, la autoimagen y el autoesquema; reduce el aislamiento social y los niveles de agresividad; disminuye la ansiedad y la depresión; mejora el rendimiento académico; trasmite reglas y normas y permite una mayor integración familiar, entre otros beneficios 7.

La actividad física es un determinante de la calidad de vida y salud, que se encuentra influido por el entorno en el que se desenvuelve la persona, el ambiente social, la cultura, los ingresos, la equidad, el sexo, la edad, las habilidades específicas y la motivación.

La OMS resalta la necesidad de trabajar en los niños y los adolescentes para que interioricen la necesidad de un estilo de vida activo y saludable que se mantenga a lo largo de su vida futura; además, recomienda que los niños y los jóvenes realicen como mínimo 60 minutos de actividad física moderada o vigorosa diariamente, combi nando actividades como caminar rápido, montar bicicleta o practicar un deporte, además de incluir ejercicios de fortalecimiento muscular y flexibilidad al menos tres veces a la semana 8.

Los gobiernos están desarrollando acciones de promoción y prescripción de la actividad física para mejorar la condición de salud de la población 9. El entorno educativo es un escenario en el que se puede hacer promoción de estilos de vida saludable con el propósito de prevenir la aparición de ECNT 10.

En atención a lo anterior, el objetivo de este estudio fue determinar la composición corporal, la aptitud física y los marcadores de riesgo bioquímico pre y post implementación de un programa de ejercicio físico en adolescentes de 10 a 17 años de un colegio público del municipio de Los Patios (Norte se Santander).

## MÉTODOS

Estudio con enfoque experimental, aleatorizado, cuya muestra estuvo constituida por estudiantes adolescentes de un colegio público de Los Patios durante el periodo de febrero a diciembre de 2020. Este estudio fue aprobado por un comité de investigación y se solicitó el asenti miento informado de los participantes y el consentimiento informado de los padres de familia de los adolescentes.

La muestra estuvo constituida por 276 estudiantes adolescentes (141 estudiantes en el grupo intervención y 135 en el grupo control) que cumplieron con los criterios de inclusión, los cuales estaban matriculados en la institución educativa durante el año electivo 2020 y no presentaban alteraciones en su estado de salud.

Los estudiantes diligenciaron una encuesta socio-demográfica. Posteriormente, se hizo la valoración de medidas antropométricas pre y post intervención: circun ferencia de cintura, circunferencia de cadera, índice de masa corporal, talla y peso, utilizando una báscula digital y un tallímetro SECA.

Se llevó a cabo la evaluación de la aptitud física según los criterios establecidos por la batería Fitnessgram® 6 para su valoración y clasificación. Los investigadores fueron los encargados de la recolección de la información y la realización de las valoraciones, las cuales se realizaron en las instalaciones del colegio, en el tiempo de clase de educación física y en presencia de los docentes de la insti tución educativa.

La intervención con el grupo de intervención se llevó a cabo mediante un programa de entrenamiento de capacidad aeróbica de intensidad moderada en forma de circuito. Los entrenamientos se realizaron cuatro veces por semana, en sesión de una hora. El entrenamiento constó de cinco momentos: calentamiento, estiramiento inicial, fase activa, estiramiento final y enfriamiento. Cada sesión se desarrolló con actividades de entrenamiento aeróbico con la supervisión de fisioterapeutas.

El entrenamiento aeróbico se llevó a cabo con ejercicio aeróbico grupal, a una intensidad de 65-75% del VO2 máximo estimado después de un periodo de adaptación a una intensidad de 50 a 60%. La intensidad del ejercicio aeróbico se ajustó para mantener el ritmo cardiaco de entrenamientos dentro de la zona prescrita, en la medida en que aumenta la aptitud cardiorrespiratoria.

Para el análisis estadístico se utilizó SPSS. Luego de la verificación de los supuestos de normalidad se concluyó que el comportamiento de las variables no tenía una distribución normal, por lo que se utilizaron pruebas no paramétricas para el contraste de hipótesis, prueba U de Mann Whitney en la comparación de dos grupos indepen dientes y prueba de rangos de Wilcoxon en la comparación de las muestras relacionadas. La correlación de la edad y las pruebas de aptitud física se llevó a cabo con el coefi ciente de Spearman. Se tomaron medidas descriptivas pre y post intervención para las variables antropométricas, laboratorios y pruebas físicas; el nivel de significación fue establecido para una p<0,05.

El estudio fue aprobado por el Comité de Bioética de la Universidad de Santander. Se contó con la aceptación voluntaria y la firma del consentimiento y asentimiento informado de los participantes. El estudio fue clasificado de riesgo minino, según la Resolución 008430 de 1993 del Ministerio de Salud de Colombia; además, esta inves tigación cumple con los cuatro principios bioéticos de investigación en seres humanos.

## RESULTADOS

La muestra estuvo conformada por 276 adolescentes de 11 a 17 años de un colegio público del municipio de Los Patios; 141 estudiantes conformaron el grupo intervención y 135 el grupo control. El grupo inter vención estuvo conformado en el 51,1% por mujeres y en el 48,9% por hombres, mientras que el grupo control estuvo integrado en el 55,6% por mujeres y el 44,4% por hombres; se evidenció que los grupos están equiparados por la variable sexo (p=0,455). (Tabla 1).

**Tabla 1:** Comparativo de los promedios observados para variables antropométricas en el grupo intervención

Edad	Sexo	n	Variables antropométricas				
			Estatura (m)	Peso (cm)	Perímetro de cintura (cm)	Perímetro de cadera (cm)	IMC
10 años	Femenino	2	Inicial: 1,60(0,04) Final: 1,60(0,04) valor p = 1,000	Inicial: 52,20(1,56) Final: 51,00(1,41) valor p = 0,180	Inicial: 64,50(0,71) Final: 63,75(1,06) valor p = 0,655	Inicial: 90,00(1,41) Final: 89,75(2,47) valor p = 0,655	Inicial: 20,52(0,30) Final: 20,05(0,33) valor p = 0,180
11 años	Femenino	10	Inicial: 1,55(0,07) Final: 1,56(0,07) valor p = 0,035	Inicial: 49,01(15,82) Final: 49,52(14,71) valor p = 0,202	Inicial: 68,10(8,18) Final: 68,45(7,94) valor p = 0,778	Inicial: 90,50(12,06) Final: 90,75(11,72) valor p = 0,875	Inicial: 20,16(5,29) Final: 20,21(5,01) valor p = 0,721
	Masculino	15	Inicial: 1,49(0,07) Final: 1,50(0,07) valor p = 0,007	Inicial: 45,11(10,27) Final: 45,32(9,08) valor p = 0,638	Inicial: 68,03(7,37) Final: 67,57(7,15) valor p = 0,080	Inicial: 82,80(8,42) Final: 82,57(8,54) valor p = 0,187	Inicial: 20,15(3,69) Final: 20,00(3,07) valor p = 0,532
12 años	Femenino	14	Inicial: 1,52(0,07) Final: 1,53(0,07) valor p = 0,004	Inicial: 46,79(10,93) Final: 46,99(10,20) valor p = 0,245	Inicial: 67,57(6,71) Final: 67,89(6,91) valor p = 0,064	Inicial: 88,00(8,54) Final: 88,14(8,37) valor p = 0,643	Inicial: 20,13(3,46) Final: 19,96(3,35) valor p = 0,177
	Masculino	18	Inicial: 1,55(0,06) Final: 1,55(0,06) valor p = 0,008	Inicial: 52,50(13,22) Final: 52,87(12,25) valor p = 0,408	Inicial: 75,28(10,88) Final: 75,11(10,78) valor p = 0,426	Inicial: 89,75(11,06) Final: 89,92(10,67) valor p = 0,388	Inicial: 21,98(5,49) Final: 21,97(5,01) valor p = 0,913
13 años	Femenino	16	Inicial: 1,58(0,04) Final: 1,59(0,03) valor p = 0,010	Inicial: 52,74(8,84) Final: 53,14(7,49) valor p = 0,453	Inicial: 69,56(7,99) Final: 69,76(7,97) valor p = 0,342	Inicial: 92,03(7,17) Final: 92,28(6,64) valor p = 0,317	Inicial: 21,12(3,45) Final: 21,10(2,99) valor p = 1,000
	Masculino	15	Inicial: 1,59(0,10) Final: 1,59(0,09) valor p = 0,103	Inicial: 52,27(12,42) Final: 52,53(11,79) valor p = 0,414	Inicial: 72,73(9,87) Final: 73,33(9,86) valor p = 0,015	Inicial: 88,93(9,98) Final: 89,03(9,91) valor p = 0,753	Inicial: 20,73(4,31) Final: 20,64(3,90) valor p = 0,730
14 años	Femenino	11	Inicial: 1,59(0,05) Final: 1,59(0,05) valor p = 0,518	Inicial: 55,11(11,05) Final: 54,45(9,88) valor p = 0,221	Inicial: 71,18(9,15) Final: 71,55(9,26) valor p = 0,130	Inicial: 93,41(8,45) Final: 93,91(8,21) valor p = 0,111	Inicial: 21,78(3,80) Final: 21,44(3,24) valor p = 0,074
	Masculino	11	Inicial: 1,59(0,09) Final: 1,60(0,09) valor p = 0,020	Inicial: 53,33(14,57) Final: 54,28(13,12) valor p = 0,131	Inicial: 73,09(10,87) Final: 73,36(11,21) valor p = 0,207	Inicial: 89,41(10,98) Final: 89,55(10,71) valor p = 0,679	Inicial: 20,88(3,98) Final: 21,03(3,51) valor p = 0,859
15 años	Femenino	8	Inicial: 1,62(0,06) Final: 1,62(0,06) valor p = 0,063	Inicial: 57,71(8,99) Final: 57,73(8,20) valor p = 0,944	Inicial: 70,88(6,38) Final: 70,44(6,16) valor p = 0,227	Inicial: 96,31(5,79) Final: 95,44(5,83) valor p = 0,055	Inicial: 22,04(2,98) Final: 21,83(2,45) valor p = 0,484
	Masculino	7	Inicial: 1,67(0,05) Final: 1,68(0,05) valor p = 0,102	Inicial: 58,81(14,05) Final: 59,16(12,54) valor p = 0,600	Inicial: 76,43(10,03) Final: 77,00(9,67) valor p = 0,176	Inicial: 94,57(7,48) Final: 94,29(6,20) valor p = 0,673	Inicial: 20,80(3,77) Final: 20,82(3,36) valor p = 0,917
16 años	Femenino	9	Inicial: 1,58(0,04) Final: 1,59(0,04) valor p = 0,026	Inicial: 46,56(6,60) Final: 48,13(6,41) valor p = 0,011	Inicial: 66,00(5,43) Final: 66,44(5,42) valor p = 0,135	Inicial: 86,61(6,39) Final: 87,28(6,25) valor p = 0,071	Inicial: 18,66(2,66) Final: 19,00(2,60) valor p = 0,110
	Masculino	1	Inicial: 1,71(0,00) Final: 1,71(0,00) valor p = N/A	Inicial: 50,60(0,00) Final: 51,80(0,00) valor p = N/A	Inicial: 68,00(0,00) Final: 70,00(0,00) valor p = N/A	Inicial: 86,00(0,00) Final: 88,00(0,00) valor p = N/A	Inicial: 17,30(0,00) Final: 17,71(0,00) valor p = N/A
17 años	Femenino	2	Inicial: 1,67(0,00) Final: 1,69(0,02) valor p = 0,317	Inicial: 50,55(9,40) Final: 51,40(9,05) valor p = 0,180	Inicial: 60,00(5,66), Final: 60,25(6,72) valor p = 0,655	Inicial: 90,00(7,07) Final: 90,75(6,01) valor p = 0,317	Inicial: 18,13(3,37) Final: 18,07(2,73) valor p = 0,655
	Masculino	2	Inicial: 1,74(0,06) Final: 1,75(0,07) valor p = 0,180	Inicial: 55,40(12,02) Final: 57,65(10,39) valor p = 0,180	Inicial: 74,00(0,00) Final: 74,50(0,71) valor p = 0,317	Inicial: 92,00(7,07) Final: 93,50(5,66) valor p = 0,180	Inicial: 18,29(2,65) Final: 18,73(1,88) valor p = 0,655

Nota. Cada celda contiene el promedio y desviación estándar para la medición pre y post intervención de cada variable en cada grupo de edad y sexo. El resultado del valor p corresponde al contraste de hipótesis mediante la prueba de Rangos de Wilcoxon.

En cuanto a las medidas descriptivas para variables antropométricas, el peso promedio en la valoración inicial en el grupo intervención fue 51,4 ± 11,78 kg, mientras que en la valoración final fue 51,77 ± 10,85 kg. En el grupo control el promedio de peso inicial fue 51,15 ± 10,69 kg. El índice de masa corporal (IMC) medio en el grupo intervención fue 20,7 ± 3,98 en la medición inicial. Para la medición final el promedio fue 20,67 ± 3,59. El grupo control exhibió un IMC promedio de 20,66 ± 3,28.

Al estratificar los resultados por variables edad y según en el grupo intervención, se pudo evidenciar un cambio estadísticamente significativo en la estatura de las niñas de 11, 12, 13 y 16 años, y en los niños de 11, 12 y 14 años (p<0,05). La tendencia del peso post intervención fue al aumento para las diferentes edades, tanto en niños como en niñas; sin embargo, se evidenció un cambio estadísticamente significativo únicamente en niñas de 16 años (p<0,05). Se registraron cambios significativos en el perímetro de cintura específicamente en el grupo de niñas de 16 años, con una tendencia al aumento (p<0,05). En los demás grupos de edad y sexo, no se evidenciaron cambios significativos en el perímetro de cintura. Igualmente, no se evidenció un cambio estadísticamente significativo respecto al perímetro de cadera y el índice de masa corporal para ningún rango de edad y sexo (p>0,05).

Se logró determinar una mediana para fuerza de empuñadura en mano derecha de 22 kg tanto para el grupo intervención como para el grupo control, mientras que para mano izquierda fue 20 y 21 kg respectivamente, sin diferencias significativas (p>0,05). La mediana de la capacidad aeróbica en el grupo intervención fue 3,2 con rango 0 a 9,1; en el grupo control fue 3,15 con rango 1,1 a 9,9, sin diferencias significativas (p>0,05) (Tabla 2).

**Tabla 2:** Medidas descriptivas pre y post intervención para pruebas físicas

Variable	Grupo	medidas descriptivas						Valor p^*^
		n	Media	ds	Mediana	Mínimo	Máximo	
Fuerza de empuñadura derecha	Intervención	131	22,60	7,20	22,00	6,00	45,00	0,860
	Control	135	22,73	7,98	22,00	9,00	50,00	
Fuerza de empuñadura izquierda	Intervención	131	21,32	7,37	20,00	9,00	44,00	0,244
	Control	135	22,28	7,38	21,00	8,00	44,00	
Promedio de fuerza de empuñadura	Intervención	131	21,96	7,03	21,00	7,50	44,50	0,596
	Control	135	22,504	7,42	22,00	9,50	47,00	
Capacidad aeróbica (YO - YO)	Intervención	133	3,48	2,12	3,20	0,00	9,10	0,358
	Control	130	3,89	1,88	3,15	1,10	9,90	
Push Up	Intervención	140	7,00	9,08	3,50	0,00	40,00	0,707
	Control	133	5,71	6,54	4,00	0,00	31,00	
Curl Up	Intervención	141	17, 91	12,97	16,00	0,00	48,00	0,805
	Control	133	17,93	14,07	15,00	0,00	62,00	
Salto alto	Intervención	130	218,06	19,45	219,00	125,00	270,00	0,171
	Control	134	215,14	19,37	212,00	131,00	265,00	
Salto largo	Intervención	131	132,95	33,61	134,00	70,00	261,00	0,039
	Control	135	124,84	30,84	120,00	55,00	209,00	
Trunk Lift	Intervención	125	24,16	8,86	25,00	6,00	40,00	0,075
	Control	120	26,279	7,80	27,00	10,00	43,00	
Sit and Reach	Intervención	117	20,18	7,72	20,00	2,00	39,00	0,996
	Control	120	20,196	7,08	20,00	2,00	36,00	

* Prueba U de Mann Whitney.

La mediana para la prueba de *Curl-Up* en el grupo intervención fue 3,5, con rango 0-40. En el grupo control este indicador fue 4, con rango 0-62. Respecto al número de abdominales, la mediana en el grupo intervención fue 16, con rango 0-62, y en el grupo control la mediana fue de 15, con rango 0-62. Ninguno de los dos indicadores presentó diferencias significa tivas entre grupos (p>0,05).

En la prueba de salto largo el grupo intervención evidenció una mediana de 134 cm, con rango 70-261 cm, mientras que en el grupo control la mediana para esta prueba fue 120 cm, con rango 55-209 cm, con evidencia de diferencias estadísticamente significativas (p=0,039).

Con relación a las pruebas de flexibilidad, el grupo inter vención en la prueba de *Trunk Lift* tuvo una mediana de 25 cm, con rango 6-40 cm, mientras que en el grupo control la mediana fue 27, con rango 10-43 cm. La prueba *Sit and Reach* arrojó una mediana para ambos grupos de 20 cm, con rango 2-39 cm. No se hallaron diferencias significativas de los resultados entre grupos en estas pruebas (Tabla 3).

**Tabla 3:** Test U de Mann Whitney para pruebas de aptitud física según sexo

FED	FEI	Promedio FE	YOYO	Push Up	Cur Up	Salto Alto	Salto Largo	Trunk Lift	Sit and Reach
Femenino: 20,38(6,39)	Femenino: 19,14(6,43)	Femenino: 19,76(6,16)	Femenino: 2,57(1,40)	Femenino: 5,73(8,41)	Femenino: 15,89(12,57)	Femenino: 213,61(16,83)	Femenino: 112,38(22,36)	Femenino: 26,35(8,34)	Femenino: 21,50(7,97)
Masculino: 24,66(7,33)	Masculino: 23,34(7,66)	Masculino: 24,00(7,22)	Masculino: 4,35(2,33)	Masculino: 8,30(9,61)	Masculino: 20,03(13,13)	Masculino: 222,12(20,88)	Masculino: 152,60(30,74)	Masculino: 21,93(8,88)	Masculino: 18,97(7,34)
valor p = 0,001	valor p = 0,002	valor p = 0,001	valor p = 0,000	valor p = 0,034	valor p = 0,056	valor p = 0,027	valor p = 0,000	valor p = 0,003	valor p = 0,056

En estudiantes entre 10 y 12 años la tendencia es la disminución del nivel de colesterol total post intervención, evidenciándose una disminución estadísticamente signifi cativa en niñas de 11 años (p<0,05).

En estudiantes de 13 a 17 años, la tendencia es el aumento del nivel de colesterol total, con evidencia de un cambio significativo en niñas de 14 y 15 años y en niños de 15 años (p<0,05). Con referencia a los niveles de triglicéridos, la tendencia en estudiantes entre 10 y 13 años es la disminución, observándose una reducción significativa del nivel de colesterol en niñas de 11 años (p<0,05). En el rango de 14 a 17 años, el nivel de colesterol tuvo una tendencia al aumento entre mediciones, aunque sin diferencias significativas respecto a la edad y el sexo (p>0,05).

De acuerdo con los resultados recolectados, se encontró una diferencia estadísticamente significativa en el grupo intervención respecto en las pruebas de flexibilidad según el sexo; se observó que la fuerza de empuñadura es mayor en hombres, así como la capacidad aeróbica, el número de flexiones, el salto alto y el salto largo (p<0,05). En la prueba *Curl Up* no se evidenciaron diferencias significa tivas respecto al sexo.

Se halló una diferencia estadísticamente significativa en los resultados observados en la prueba *Trunk Lift,* donde las mujeres exhibieron mayor flexibilidad que los hombres (p<0,05). También se observó dicho compor tamiento para la prueba *Sit and Reach,* pero no se pudo concluir que existiera una diferencia estadísticamente significativa respecto al sexo (p>0,05).

Se evidenció una correlación directa y significativa entre los resultados para las pruebas de aptitud física *Push Up, Curl Up,* salto largo y fuerza de empuñadura, con la edad de los estudiantes (p<0,05); es decir, a mayor edad mejor capacidad física para estas pruebas. En la prueba de flexibilidad *Trunk Lift* se encontró un coeficiente de corre lación negativo (r-0,032), lo cual infiere una pérdida paulatina de la flexibilidad a medida que aumenta la edad.

## DISCUSIÓN

Los resultados obtenidos al analizar las características antropométricas evidencian que los niños y las niñas presentan un IMC en peso saludable, que en niños es de 17,3-21,98 y en las niñas de 18,7-22,04; resultados similares se evidenciaron en el estudio realizado por Rodriguez (11) en adolescentes de Bogotá, en el cual se encontró que los niños tenían un IMC de 19 y las niñas de 20. En contraste, se obtuvieron resultados opuestos en el estudio realizado por Pomar y Bahamon en el 2018, en el cual los niños presentaron un mayor IMC y porcentaje de grasa. Igualmente, en el estudio llevado a cabo en Floridablanca por Sanchez 12 se encontró que casi la quinta parte de los participantes presentó un IMC que no es saludable de acuerdo con los criterios de la batería Fitnnesgram 13.

La relación cintura/cadera refleja que las niñas tienen menor riesgo cardiometabólico en comparación con los niños; estos resultados difieren con los encontrados en un estudio en el cual las niñas presentaron un mayor perímetro de circunferencia de cadera (83 cm) que los niños (80 cm). En el estudio de Galán-López 14 la circunferencia de los niños fue significativamente mayor que la de las niñas.

La evaluación de potencia de los adolescentes evidenció un registro de salto largo en las niñas de 111,75 ± 21,61 cm y en los niños de 147,53 ± 32,02 cm; resul tados similares se reportaron en el estudio de Gamardo 15 , en el cual los niños registrarón 149,1 ± 25,9 cm, lo cual refleja que los niños presentan mayor potencia en miembros inferiores. Por su parte, en el estudio Fuprecol 16 se encontró un comportamiento diferente: 139,2 ± 31,1cm para los niños y 111,0 ± 22,2 cm para las niñas.

La intervención con el grupo intervención se llevó a cabo por medio de un programa de entrenamiento de capacidad aeróbica de intensidad moderada en forma de circuito. Los entrenamientos se hicieron cuatro veces por semana, en sesión de una hora. El entrenamiento constó de cinco momentos: calentamiento, estiramiento inicial, fase activa, estiramiento final y enfriamiento. Cada sesión se desarrolló con actividades de entrenamiento aeróbico y la supervisión de fisioterapeutas, lo cual contribuyó a que se observaran cambios significativos en las variables estudiadas como peso y perímetro de cintura. En contraste, en un estudio realizado por Ardoy *et al.*^17^, la inter vención no produjo cambios significativos en las variables antropométricas y de composición corporal estudiadas: peso, estatura, índice de masa corporal, sumatorio de seis pliegues, porcentaje graso, índice de masa grasa, perímetro de cintura, relación cintura/estatura, masa libre de grasa e índice de masa libre de grasa. La capacidad aeróbica ha mostrado una relación muy estrecha con la salud cardiovascular en niños y adolescentes.

En un estudio realizado por García Hermoso 18 se encontraron diferencias entre la línea base y el tercer año en solo dos parámetros cineantropométricos: talla (p=0,036) y zIMC (p=0,015). Respecto a los parámetros metabólicos, se observaron diferencias en el CT entre la línea base y el tercer año (p=0,049); en el colesterol LDL entre diversos momentos temporales (línea base > segundo año, p=0,048; línea base > tercer año, p=0,049; primer año >2° año, p=0,043; primer año > tercer año, p=0,041); en la glucosa entre la línea base y el tercer año (p=0,002); y, por último, en el índice CT/HDL entre la línea base, el segundo y el tercer año (p=0,018 y p=0,049, respectivamente. No se observaron cambios en el estado puberal los hábitos alimentarios o la actividad física diaria. En el presente estudio se observaron diferencias entre la línea base y el primer año: el nivel de colesterol total medio en el grupo intervención en la medición inicial fue 151,4 ± 61,34 mg/dL, promedio que para la medición final ascendió a 162,78 ± 34,87 mg/dL. En cuanto al nivel de triglicéridos, en la medición inicial en el grupo intervención fue 102,48 ± 40,69 mg/dL, y se observó un comportamiento similar para la medición final, ya que se determinó un promedio igual a 102,15 ± 39,48 mg/ dL. En contraste, en el estudio realizado por Alfredo Cordova 19, el peso corporal, el índice de masa corporal, la circunferencia de cintura, los trigliceridos y el colesterol disminuyeron en forma significativa con la práctica de ejercicio físico de forma regular.

En un estudio llevado a cabo por Valero 11, el desempeño muscular fue evaluado con las pruebas de salto vertical, salto longitudinal y fuerza prensil, en las cuales los participantes presentaron un bienestar físico más saludable en los indicadores, con un valor p<0,001, mientras que en el actual estudio el valor de p para la prueba de fuerza prensil fue de p<0,059 y en salto vertical p<0,17 con diferencias estadísticamente significativas en el salto largo con un valor p<0,039.

En la presente investigación se pudo constatar que a mayor edad del escolar había una mejor respuesta en pruebas de aptitud física tales como Push-Up y *Curl-Up,* similar a lo encontrado en un estudio realizado por Martinez y Ceballos 20, en el cual se reportaron cambios significativos en dichas después de un entrenamiento de fuerza realizado durante 16 semanas.

En un estudio llevado a cabo por Torres y Galeano 21, que evaluó los efectos de un programa de entrena miento sobre fuerza y flexibilidad, se reportaron cambios estadísticamente significativos en la flexibilidad luego de la intervención con un programa de ejercicios estructu rados aplicado por 12 semanas. Este estudio difiere de lo encontrado en la presente investigación, en la cual se observó una pérdida paulatina de la flexibilidad a medida que aumenta la edad.

En conclusión, se reportó aumento de peso y de perímetro de cintura en niñas de 16 años; no se observaron cambios estadísticamente significativos en el perímetro de cadera e índice de masa corporal para ningún rango de edad y sexo.

En relación con los marcadores bioquímicos, en los escolares de 10 a 13 se evidenció una disminución del colesterol total y triglicéridos, mientras que en aquello de 14 a 17 años, el nivel de colesterol tuvo una tendencia al aumento entre mediciones, aunque sin diferencias signi ficativas respecto a la edad y el sexo.

Se encontró asimismo que la capacidad aeróbica y la fuerza de empuñadura es mayor en hombres, en la prueba *Trunk Lift* y *Sit and reach* las mujeres exhibieron mayor flexibilidad que los hombres.

Los hombres presentaron mayor rendimiento físico en las pruebas de Push-Up, *Curl-Up,* salto alto, salto largo; se evidenció que a mayor edad hay una mejor capacidad física para estas pruebas.

La práctica regular de ejercicio físico en edades tempranas y en los diferentes entornos en los que se desenvuelven los niños y los adolescentes constituye un factor protector de la salud que genera un impacto positivo en la calidad de vida de la población.

Los autores concluyen que es importante evaluar la condición física de los escolares como estrategia para la prevención de enfermedades crónicas no trasmisibles. A su vez, se recomienda que se promueva la práctica regular de actividad física en el entorno escolar y en el hogar, estimulando las diferentes cualidades físicas básicas y teniendo en cuenta la edad y el nivel de desarrollo de los escolares. Además, es importante que estas actividades se realicen con un componente lúdico para lograr una mayor adherencia por parte de los escolares.
